# Urinary Continence Following Repair of Intermediate and High Urogenital Sinus (UGS) in CAH. Experience with 55 Cases

**DOI:** 10.3389/fped.2014.00067

**Published:** 2014-07-02

**Authors:** Maria Marcela Bailez, Estela Susana Cuenca, Victor Dibenedetto

**Affiliations:** ^1^J. P. Garrahan Children’s Hospital, Buenos Aires, Argentina

**Keywords:** urogenital sinus mobilization, intermediate urogenital sinus, high urogenital sinus, urinary continence after urogenital reconstruction, congenital adrenal hyperplasia

## Abstract

**Aim:** To evaluate postoperative urinary continence in patients with congenital adrenal hyperplasia (CAH) with intermediate (IT) and high urogenital sinus (UGS) who underwent a UGS mobilization maneuver.

**Methods:** We called IT to those that although needing an aggressive dissection to get to the vagina, still have enough urethra proximal to the vaginal confluence. Very low variants are excluded from this analysis. Dissection always started in the posterior wall of the UGS with an aggressive separation from the anterior rectal wall. If the wide portion of the vagina was reached dissection stopped and the UGS opened ventrally widening to the introitus. Nineteen patients were treated using this maneuver (Group 1). When more dissection was required the anterior wall of the UGS was dissected and carefully freed from the low retropubic space. Then the UGS was opened either ventrally or dorsally. Thirty three patients required this approach (Group 2). Combined procedures were used in three patients with high UGS (Group 3).

**Results:** Mean age at the time of the repair and length of the UGS were 12.2 years (4 months–18 years) and 3.75 cm (3–8 cm) for G1; 8 years (5 months–17 years) and 6.34 cm (4–12 cm) in G2 and 8.3 years (2–14 years) and 11.5 cm (11–12 cm) in G3. All patients had been regularly followed. Mean age at last follow up was 14.3, 17, and 9.9 years for Groups 1, 2, and 3, respectively. All patients continue to void normally and are continent. All patients have two separate visible orifices in the vulva. Only three are sexually active.

**Conclusion:** Urogenital sinus mobilization for vaginoplasty in girls with CAH does not compromise voiding function or urinary continence.

## Introduction

The description of the technique of total urogenital mobilization (TUM) by Peña in 1997 (1) prompted us to apply the same principle to the correction of other forms of urogenital sinus (UGS) such as that found in congenital adrenal hyperplasia (CAH) (2). We have applied the TUM technique to cases in which the urethra proximal to the confluence, as evaluated by contrast studies had adequate length. The advantage of this technique is that it avoids the often difficult separation of the urethra from the vagina. Some authors have raised concerns about a possible adverse effect the TUM could have on future urinary continence. Here, we report the results regarding urinary continence in patients who were toilet trained at the time of surgery or who have become old enough to have sphincter control.

## Materials and Methods

**Figure 1:**
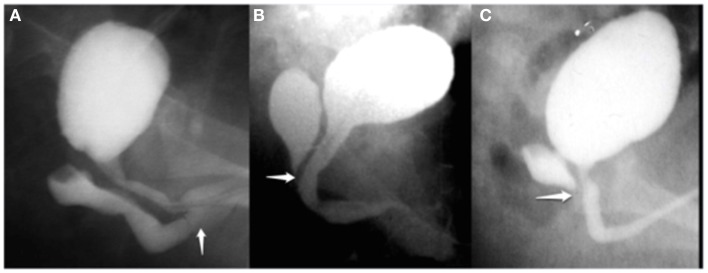
**Genitography is very useful for the study of vaginal morphology, dimension, and relation to the urethra**. Depending on vaginal confluence in the UGS was classified in **(A)** low, **(B)** intermediate, and **(C)** high variant. The white arrows show the confluence of vagina in the UGS.

Retrospective chart review of patients with CAH operated at our institution with intermediate and severe form of UGS since October 1996 that have been followed until they were toilet trained. UGS were classified as low, intermediate, and high according to the point of confluence of the urethra and vagina based on a contrast genitogram (Figure 1). Very low variants of UGS could be corrected with a simple operation and are not included in this report. Low variants (Group 1) could be repaired with aggressive dissection of the posterior wall of the UGS and vagina from the rectum as proposed in redo surgery for vaginal stenosis (3). Intermediate forms (Group 2) required aggressive posterior, lateral, and anterior mobilization to bring the urethra and vagina down to the perineum. In the high cases (Group 3) in addition to the perineal mobilization, we performed separation of the urethra from the vagina through the anterior anorectal approach in a prone position (modified ASTRA).

Urinary continence was evaluated during the clinical follow up visits by asking parents and children about their voiding habits. Patients who have not reached toilet training age were excluded.

### Surgical technique

**Figure 2:**
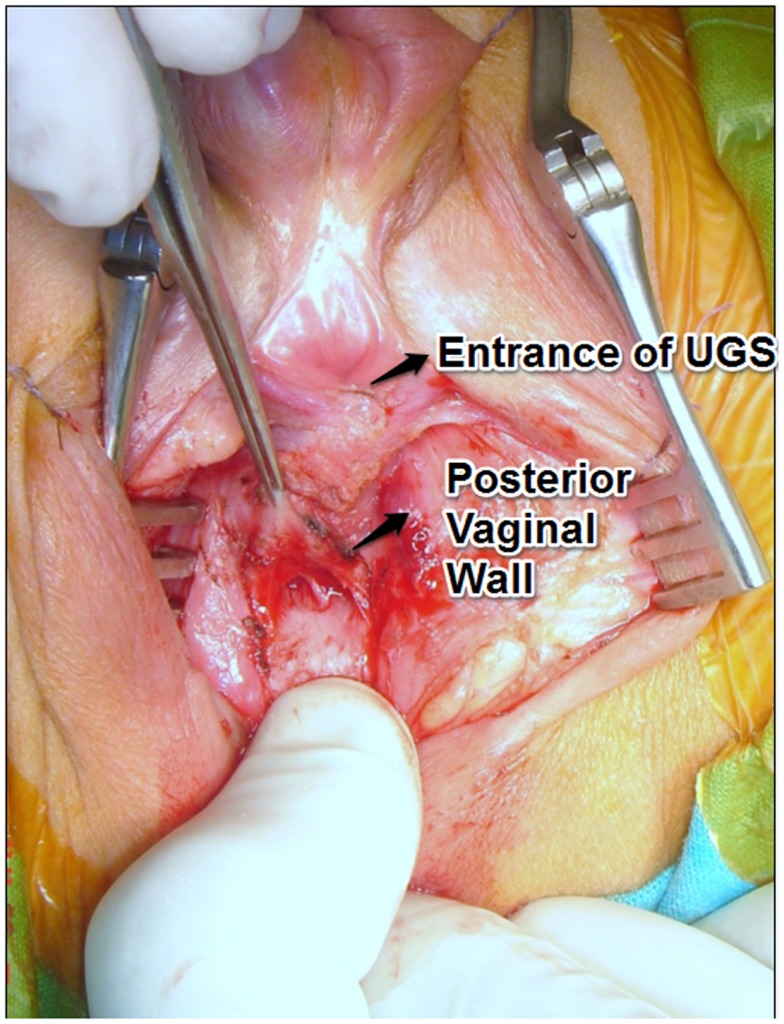
**Dissection of the posterior vaginal wall, separating it from the rectal wall before sectioning the UGS**. A rectal finger is very useful to facilitate vaginal exposure.

All operations were started in the dorsal lithotomy position. The UGS was dissected circumferentially without opening its walls. The posterior dissection was done first separating the posterior wall of UGS and vagina from the rectum (Figure 2). All the length of the UGS is preserved since it may be needed at the end for the vaginoplasty. The dissection along the lateral aspects of the sinus was done next. At this point, if the wide portion of the vagina could be reached the dissection stopped and the UGS was opened ventrally widening the introitus with either a flap of the UGS or a skin flap.

**Figure 3:**
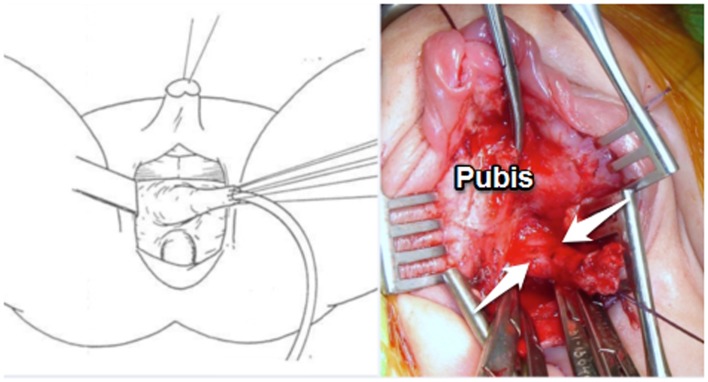
**“Total urogenital mobilization”: TUM in lithotomy position in an intermediate UGS**.

When more dissection was required, the anterior wall of the UGS was dissected and carefully freed from the low retropubic space (Figure 3). This maneuver facilitates the exteriorization of the previously dissected posterior vagina. At this point, all the UGS walls had been dissected. The UGS was opened either ventrally or dorsally if a Passerini flap was preferred for the anterior vaginal wall depending on the urethral position. The mobilized vaginal walls were sutured to the perineal skin.

In patients with a high UGS, these maneuvers were combined with the principles of pull-through (placing a balloon catheter in the vagina endoscopically and keeping part of the distal UGS as urethra). After dissection of the UGS in the lithotomy position, the patient was turned prone and approached using a modified ASTRA principle (4). We used an extended perineal approach in the prone position but rectal wall was not incised (EPPA for extended prone perineal approach).

**Figure 4:**
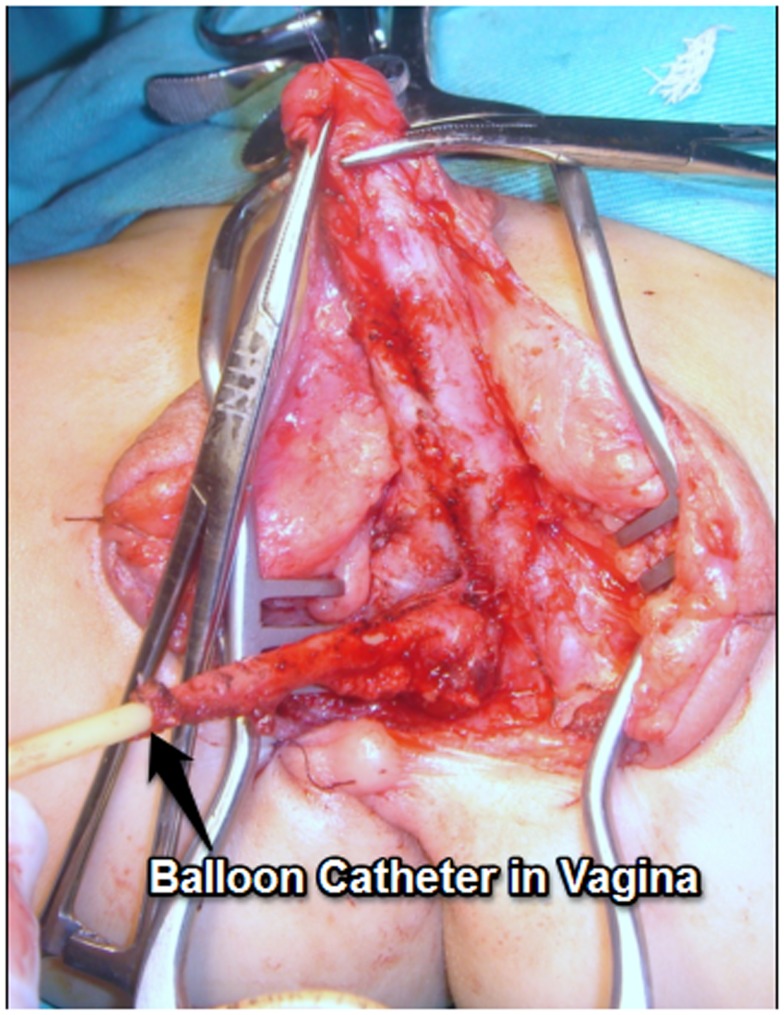
**Mobilization the UGS in the lithotomy position without opening it in a high variant**. A balloon catheter has been previously inserted in vagina.

**Figure 5:**
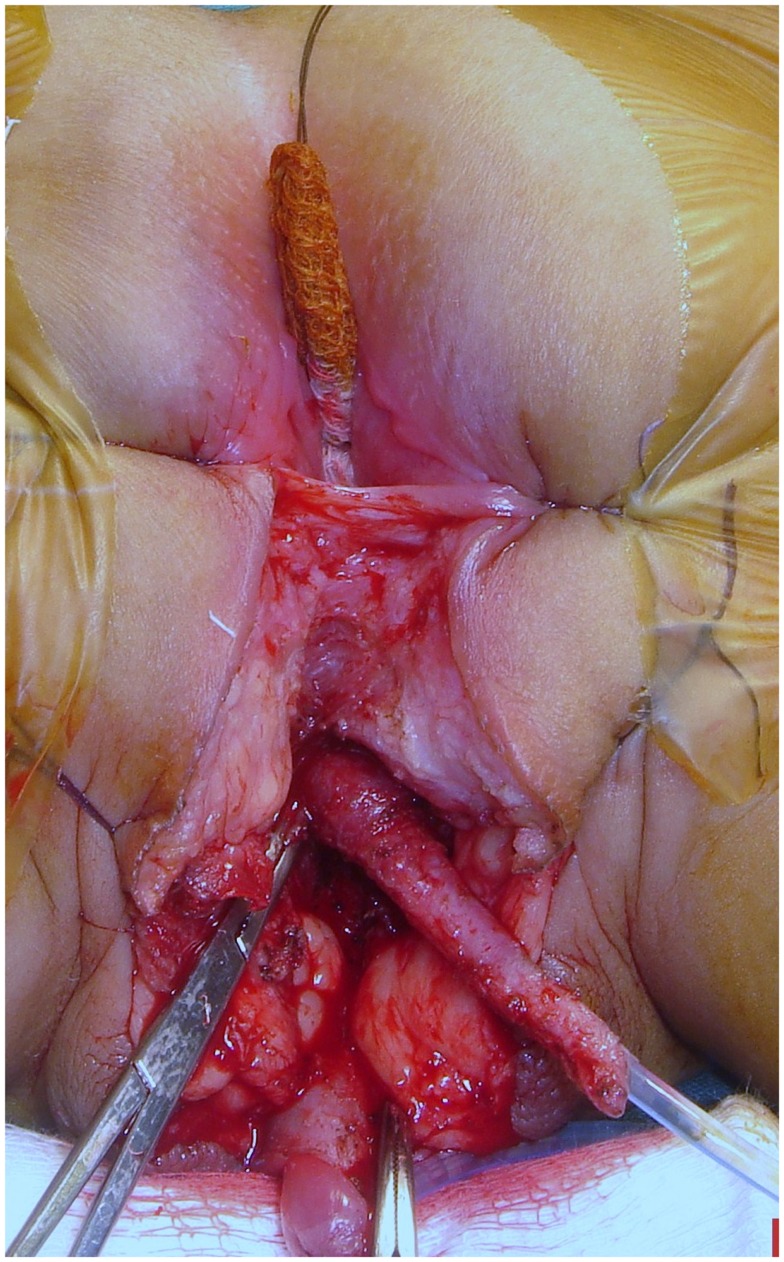
**Prone position**. The modified anterior sagittal transrectal approach (ASTRA). Note that the balloon catheter was previously inserted in vagina. The midline perineal incision is extended around the anocutaneous junction between 4 and 8 h to get better exposure. The rectum is retracted using sutures and a retractor to avoid opening the anterior rectal wall as proposed in the ASTRA technique.

**Figure 6:**
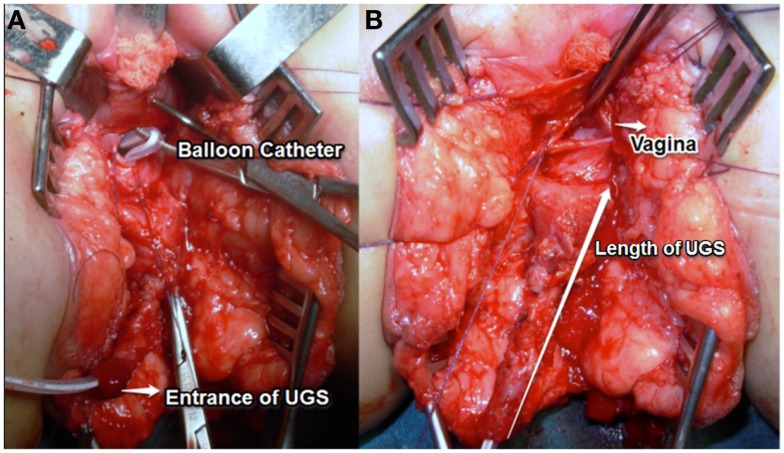
**(A,B)** The vagina opened over the balloon, the foley catheter repositioned in the proximal urethra. The long arrow shows the length of the UGS.

**Figure 7:**
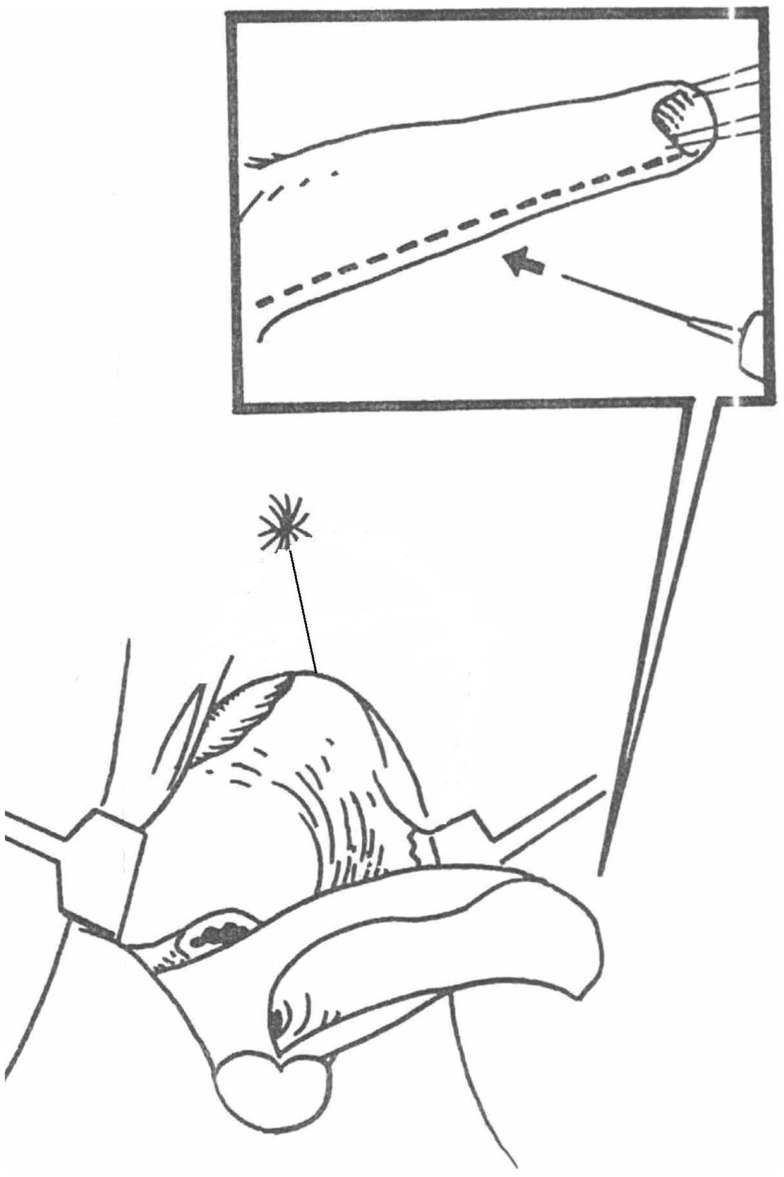
**Diagram of the UGS transected ventrally (patient is in prone position) and everted to reach the anterior wall of the vagina**. In this way, the proximal part of it stays as urethra.

**Figure 8:**
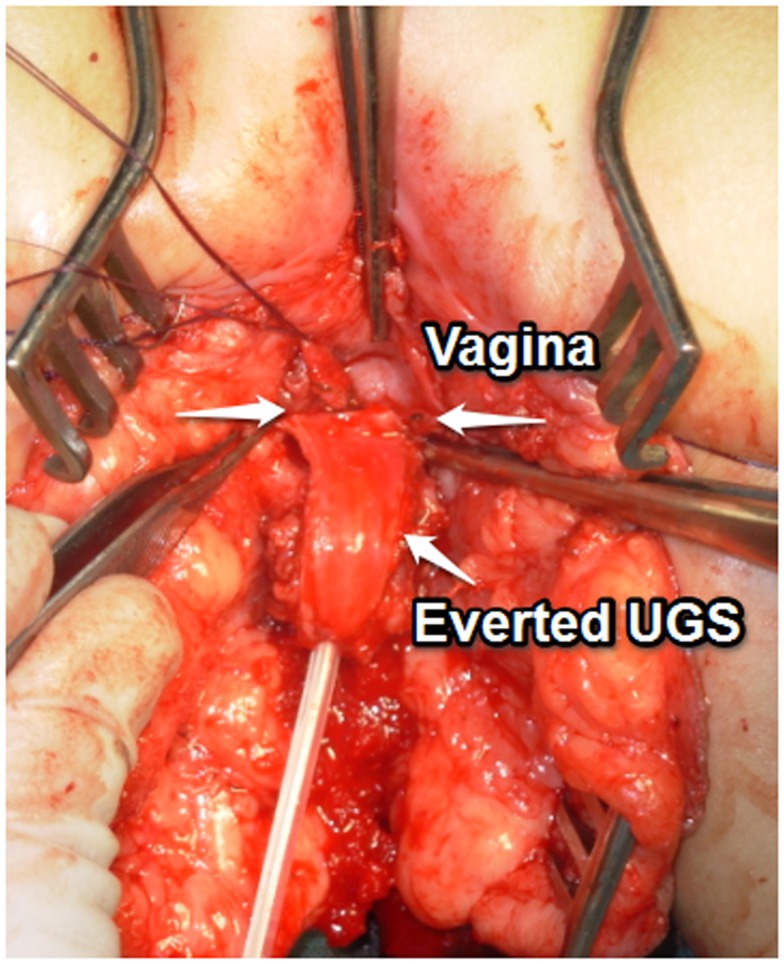
**UGS everted to reach the anterior vaginal wall**.

We start with placing a balloon catheter (most of the times a Foley catheter inserted over a guide wire under endoscopy). We mobilize the UGS in the lithotomy position without opening it (Figure 4). We then turn the patient to the prone position (Figure 5). The midline perineal incision is extended around the anocutaneous junction between 4 and 8 h to get better exposure. The rectum is retracted using sutures and a retractor to avoid opening the anterior rectal wall as proposed in the ASTRA technique. The vagina is opened over the balloon, the Foley catheter repositioned in the proximal urethra, and the urethrovaginal fistula closed, taking care not to denervate the bladder neck (Figures 6A,B). The short vagina (which is usually the case in these patients) needs to be exteriorized. The transected perineal skin between the vagina and rectum is used to reconstruct the dorsal wall and the previously dissected UGS for the ventral wall. For that purpose, it is opened ventrally (considering the prone position but it is the dorsal wall of the UGS) and everted to reach the vagina. In this way, the proximal part of it stays as urethra (Figures 7 and 8).

The length of the UGS was measured with a ruler from its external opening to the onset of the vagina during surgical reconstruction.

## Results

In 19 patients, the posterior and lateral dissection was sufficient to correct the malformation (Group 1). The mean age at the time of surgery was 12.2 years (4 months–18 years). The mean age at last follow up in this group was 14.3 years. All these girls are toilet trained and continent. The length of the UGS in this group was 3.75 cm (range 3–8 cm).

In 33 patients, retropubic dissection was also needed to complete the correction (Group 2) and toilet trained. The mean age at the time of surgery was 8 years (5 months–17 years). The mean age at last follow up for Group 2 was 17 years and all void normally. The length of the UGS in this group was 6.34 cm (range 4–12 cm).

Three patients required an additional ASTRA approach with preservation of part of the UG sinus as urethra (Group 3). The mean age at the time of surgery was 8.3 years (2–14 years). The mean age at last follow up was 9.9 years. They are all toilet trained.

All patients continue to void normally and are continent. Of the 55 patients, only 3 have become sexually active. They refer a satisfactory function (Table 1).

**Table 1:** **Results**.

Group	Number patients	Age at surgery	Length UGS	Mean actual age (years)
1	19	12.2 years (4 months–18 years)	3.75 cm (3–8 cm)	14.3
2	33	8 years (5 months–17 years)	6.34 cm (4–12 cm)	17
3	3	8.3 years (2–14 years)	11.5 cm (11–12 cm)	9.9

## Discussion

Urogenital sinus abnormalities are a spectrum that goes from a labial fusion to an absent vagina depending on the location of the vaginal confluence in the UGS. Powell described four types: I: labial fusion. II: distal confluence. III: proximal confluence. IV: absent vagina (5). In 1969, Hendren described different procedures required for reconstruction depending on the location of the vaginal confluence in the UGS related to the external sphincter (low when distal and high when proximal to the sphincter (6). This has been very helpful for reconstructive understanding but the vagina is not always high or low and the sphincter not well seen. We have found vaginas located from a normal position to an entrance in the bladder in a wide spectrum.

However, others have recently disputed this concept maintaining that although the relationship of the confluence to the sphincter may be variable in cases of UGS not related to virilization, in CAH the urethra above the confluence is always normal (7, 8).

Classically, cases of low confluence were repaired by a “flap vaginoplasty” (9) and the mid to high by a “pull-through vaginoplasty” (10).

**Figure 9:**
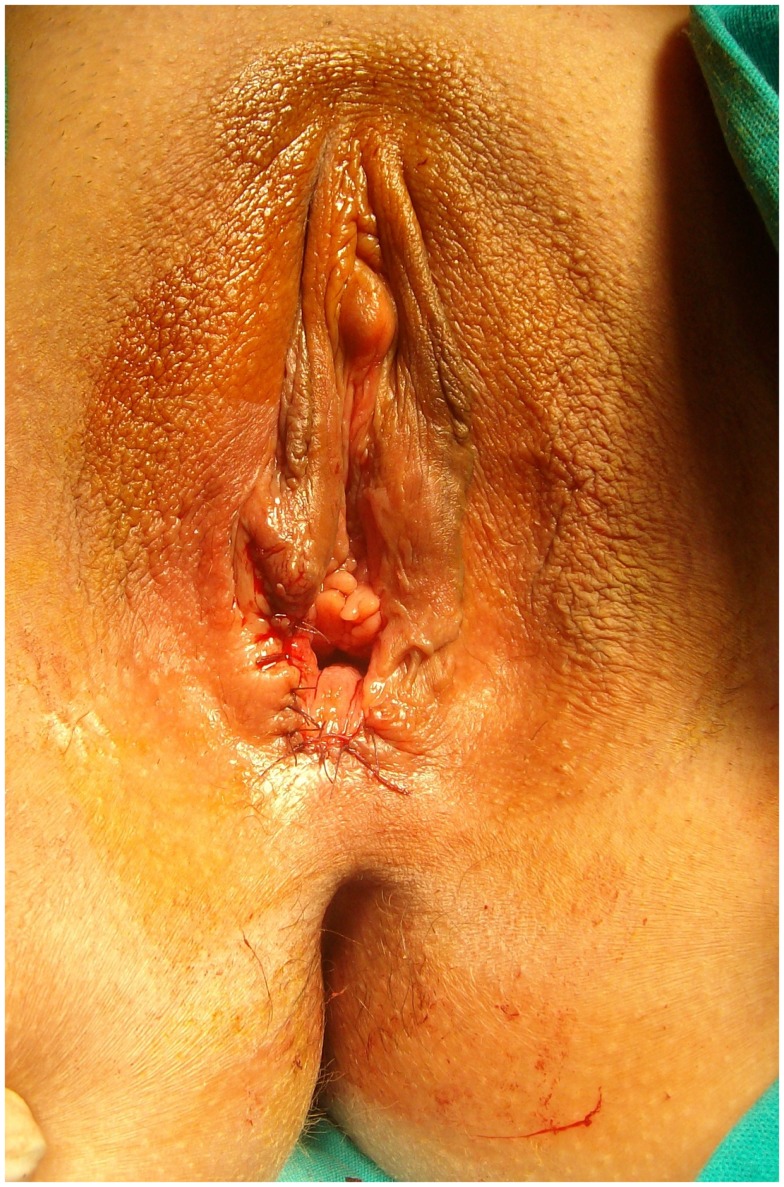
**Postoperative aspect of a vaginoplasty after an aggressive dissection of the posterior vaginal wall bringing the mucosa out to a wide perineal flap**.

However, even in the low type, we advocate a very aggressive dissection of the posterior vaginal wall, separating it from the rectal wall. The vagina is then cut in the midline well back into its normal caliber and at this point a wide cutaneous flap can be sewn using delicate sutures (Figure 9). A rectal finger is very useful to facilitate vaginal exposure (Figure 2). This maneuver of bringing the vagina out rather than skin prevents the known complications of the Fortunoff flap applied without vaginal mobilization (growing of hair and stenosis).

Exteriorization of the high vagina in the severely masculinized female is a surgical challenge: we prefer to detach the vagina from the UGS and then connect it to the perineum. The pull-through principle consists in placing a balloon catheter into the vagina endoscopically to allow locating it by palpation deep in the perineum during the dissection. The UGS is approached like a bulbous urethra. Perineal anatomy in severe cases is like that a normal male. The vagina is incised over the balloon, detached from its entry point in the UGS, and the anterior wall carefully dissected off the overlying urethra (Figures 6A,B). The vaginal walls to reach the perineum may be constructed using a combination of inverted U cutaneous flap (Fortunoff), preputial flap (11), and redundant tissue from the UGS (12). The anterior sagittal transrectal approach in a prone position (ASTRA) is another way of exposure of the high UGS (4) although some consider this approach unnecessary in primary cases (13).

Between these extremes, we found what we called intermediate UGS. Although the vaginal opening is far from the UGS opening, there is enough proximal urethra to avoid urethra vaginal dissection and separation. After Peña’s description of “TUM” for the treatment of cloacas in 1997 (1), we (14) and others (15) started using this maneuver to treat intermediate UGS abnormalities. The UGS is mobilized in block from the perineum. We use it in the lithotomy position, mobilize it without previous opening to prevent bleeding and never amputate the sinus tissue until the end as it may be used in the repair (Figure 3). Each patient must be individualized and this technique can be combined with a pull-through if required but it has simplified many of these repairs.

Rink described a variant that he calls partial urogenital mobilization (PUM) (16), stopping dissection at the level of the pubourethral ligament. Currently regardless of the level of the confluence, he starts with PUM, allowing a unified approach to all repairs. Some consider PUM a misinterpretation of the term TUM in that TOTAL refers to en-block mobilization of the urethra and vagina and not to the extent of proximal dissection (8).

A posterior sagittal approach has been also described to correct intermediate and high UGS (4, 17). In our opinion, the best way to treat the high UGS is the combination of techniques. We start with placing a balloon catheter (most of the times a Foley catheter inserted over a guide wire under endoscopy). We mobilize the UGS in the lithotomy position without opening it (Figure 4). We then turn the patient to the prone position. The rectum is retracted and sometimes the anterior wall opened. The vagina opened over the balloon, the Foley catheter repositioned in the proximal urethra and the uretrovaginal fistula closed, taking care not to denervate the bladder neck (Figure 6). The short vagina (which is usually the case in these patients) needs to be exteriorized. The transected perineal skin between the vagina and rectum is used to reconstruct the dorsal wall and the previously dissected UGS for the ventral wall. For that purpose, it is opened ventrally and everted to reach the vagina. In this way, the proximal part of it stays as urethra (Figures 7 and 8).

Thus in Group 3, we combined the pull-through, TUM, and ASTRA principles instead of using a single technique.

Thus in our experience, the technique utilized to correct UGS in CAH needs to be tailored to the individual anatomy of the patient. The operations we performed have not compromised urinary continence as also previously observed by others (18). The division into groups presented here depends on the operation needed to correct the UGS.

Our study has some limitations. In the first place, it is retrospective. We also estimated that separation of the urethra and vagina was necessary in some cases based on the length of the urethra but we did not measure the urethra endoscopically. Finally, voiding function and continence were determined by interviews without objective measurement. Nevertheless, we and our patients and families have been stratified with the medium term results. Longer follow up till onset of sexual activity is necessary to evaluate the adequacy of the vaginoplasty.

## Conclusion

Combination of strategies including a careful but complete dissection of the UGS, let us end up with two visible orifices in the perineum without clinical impairment of urinary function in cases of CAH. More time is needed for other functional results.

## Conflict of Interest Statement

The authors declare that the research was conducted in the absence of any commercial or financial relationships that could be construed as a potential conflict of interest.
